# Triboelectric charge saturation on single and multiple insulating particles in air and vacuum

**DOI:** 10.1038/s41598-023-42265-0

**Published:** 2023-09-13

**Authors:** Reuben D. Cruise, Stanley O. Starr, Kathryn Hadler, Jan J. Cilliers

**Affiliations:** Department of Earth Science & Engineering, Imperial College London, London, UK

**Keywords:** Electronic properties and materials, Applied physics, Techniques and instrumentation

## Abstract

Triboelectric charge transfer is complex and depends on contact properties such as material composition and contact area, as well as environmental factors including humidity, temperature, and air pressure. Saturation surface charge density on particles is inversely dependent on particle size and the number of nearby particles. Here we show that electrical breakdown of air is the primary cause of triboelectric charge saturation on single and multiple electrically insulating particles, which explains the inverse dependence of surface charge density on particle size and number of particles. We combine computational simulations with experiments under controlled humidity and pressure. The results show that the electric field contribution of multiple particles causes electrical breakdown of air, reducing saturation surface charge density for greater numbers of particles. Furthermore, these results show that particles can be discharged in a low pressure environment, yielding opportunities for improved industrial powder flows and dust mitigation from surfaces.

## Introduction

Electrical charge can be exchanged when two materials contact. This process is called triboelectric charging, and it is prevalent in many processes involving the contact of insulating materials, giving rise to a number of problems and opportunities. A key area affected by triboelectric charge build-up is that of industrial powder flow processes. Charge build-up can lead to increased friction and hence energy losses in such flows, reducing powder flowability^1,2^. In addition to reducing flowability, this charge build-up can cause clogging of fluidised bed reactors^3–7^ and pneumatic conveyers^8,9^. This agglomeration and segregation in powder flows causes problems for industrial products, leading to non-uniform dosage in pharmaceutical products for example^10^. Large charge build-up causes the creation of strong electric fields which can cause electrical discharge and arcing. Such arcing can cause damage to electronic devices^11,12^ and pose a significant fire and explosion hazard^13,14^.

Triboelectric charge build-up can be harnessed for a number of applications despite the negative impact it has on some processes. These applications include toner particle charging for electrophotography^15^, electrostatic mineral separation^16–21^, and electrodynamic dust shields for solar farms^22^ and lunar exploration^23,24^. A better knowledge of triboelectric charging can improve the understanding of a number of natural processes, in addition to enabling new technological applications. Such natural processes include particle behaviour in dust storms^25–28^, electrification of volcanic plumes^29,30^ which leads to levitation of ash into the Earth’s ionosphere^31^, and the aggregation of dust particles in planetary formation^32–34^.

There are still many outstanding questions about which mechanisms dominate the behaviour of triboelectric charge transfer and saturation. Theory development and experimentation on the triboelectric charging of single particles under controlled conditions have been performed to understand these charge transfer mechanisms better. Experiments on the charge transferred per contact show that particles charge towards a saturation level in a manner similar to a capacitor, and that the rate of charging can be dependent on factors such as impact velocity and angle^35–38^. Particle saturation charge has been shown to be proportional to particle size^39^, but the surface charge density reduces with increasing particle size^40^.

The mechanism for charge saturation on groups of particles in close proximity is still not well understood. Increasing the number of particles in a mixture reduces the charge per particle at saturation^41,42^. Surface potential altering^43^ and dielectric breakdown of air^44^ are the two primary mechanisms available for explaining this reduction in charge per particle. In the surface potential altering model, the electric field created by additional particles induces a charge in a conducting charging surface. This charge induction may alter the work function of the charging surface, in turn affecting the final saturation charge attainable by particles that contact it^40,43,45^. In the breakdown model, the electric field created by a particle or group of particles may become strong enough to cause the dielectric breakdown of the surrounding medium. This phenomenon has been considered as a saturation mechanism for single particle charging^46,47^ and the formation of nano-scale oppositely charged patches on the surface of materials^48^. It has also been proposed as a potential cause for reduction in charge saturation on larger groups of particles relative to a single particle, however this has yet to be definitively demonstrated^44,49^.

In this work, we determine whether the charge saturation mechanism for the triboelectric charging of multiple identical insulating particles is electrical breakdown. We describe a new method of simultaneous triboelectric charging and charge measurement. Theory for the charge saturation on single particles is discussed, then verified experimentally in a controlled humidity and temperature environment and under vacuum. The theory and experiments are then extended to multiple particles, using a combination of experimental results and simulations of the electric field build-up on multiple particles to determine the mechanism of charge saturation. Finally, we test the effect of pressure on saturation charge, resulting in a new method for electrostatic charge mitigation which can be applied to dust removal.

## Results

### Experimental development

**Figure 1:**
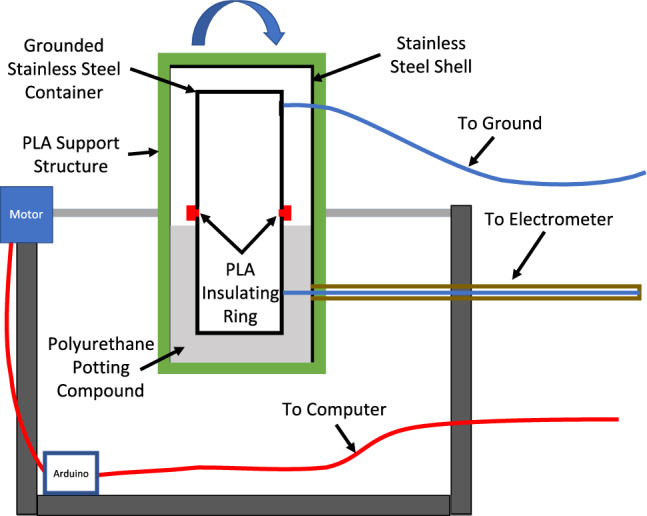
Schematic representation of the automated simultaneous triboelectric charging and charge measurement system. A charging capsule is made by attaching the inside of a Faraday cup to an identical grounded container separated by an insulating ring. The capsule is enclosed in a grounded shell for electromagnetic shielding. The system is rotated using a servo motor controlled by an Arduino. Charge in the Faraday cup is measured using an electrometer.

An automatic charging system was designed that can triboelectrically charge particles up to their saturation charge while simultaneously measuring their charge build-up over time. A schematic of this system is shown in Fig. 1 and it has been described in another work^38^. The charge on a particle can be measured using a Faraday cup connected to a coulombmeter. Here we used a Faraday cup as one of the triboelectric charging surfaces. An identical stainless steel container to the inner cup of the Faraday cup was attached to the top of the Faraday cup, using a 2 mm thick 3D printed PLA ring to electrically insulate the two cups from each other, in a manner similar to Schella et al.^42^. This created an enclosed charging capsule, one half being the inside of a Faraday cup, the other half being an identical grounded stainless steel container. The charging capsule was then enclosed in a grounded stainless steel outer shell, serving as an electromagnetic shield to avoid background noise in charge measurements. The entire system was then mounted on a servo motor, controlled using an Arduino microcontroller so that it rotates 180° back and forth around the horizontal axis. Two 5 mm holes were drilled in the side of the shell and capsule to allow the air to vent when the system is placed in a vacuum. In addition, the capsule is detachable to allow the addition and removal of particles and cleaning of the charging surfaces. We used a Keithley 6517b electrometer which is fit with a coulombmeter for charge measurements.

**Figure 2:**
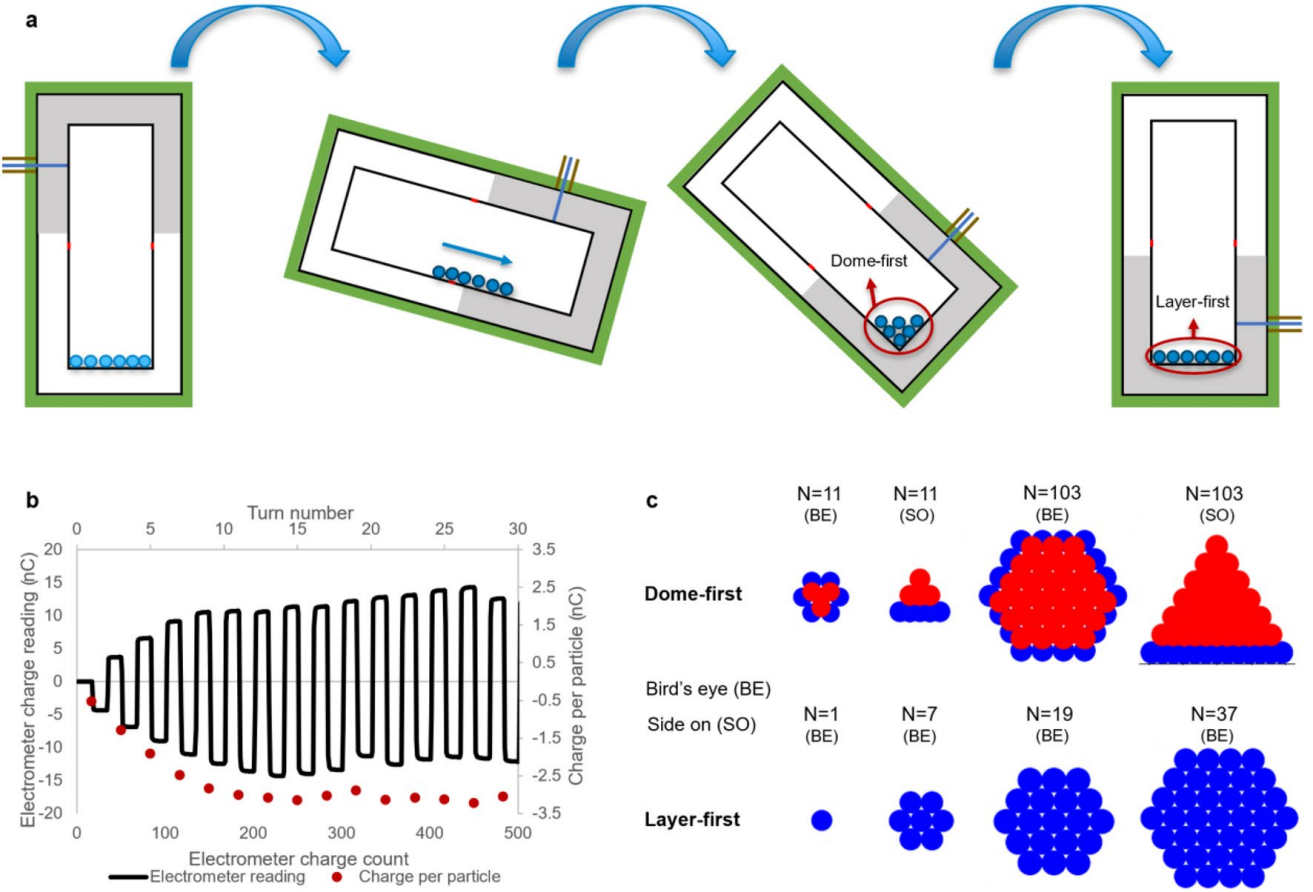
Simultaneous triboelectric charging and charge measurement, outlining stages of maximum packing density and electrostatic adhesion. (**a**) Schematic (not to scale) of the charging capsule from Fig. 1 rotating with multiple particles contained inside. As particles move within the capsule, they gain charge triboelectrically. The strongest local electric field is created when the particles agglomerate in the corner of the capsule during rotation, this packing is labelled dome-first. Here, the sum of distances between the particles is minimised, maximising the cumulative electric field which is inversely proportional to distance from charge sources. When the particles form a layer on the bottom of the capsule after rotation, the strongest normal electric field is created for each particle, this packing is labelled layer-first. (**b**) The charge measurement on the electrometer changes when the particles pass into or out of the Faraday cup half of the capsule, yielding a square wave signal. The charge per particle is calculated as the amplitude of the square wave divided by the total number of particles. (**c**) A 3D representation of the dome- and layer-first geometries generated using Python and explained in the section on multiple particle electric field calculations. The blue spheres represent the base layer of particles, while the red spheres are additional layers built up in the dome-first model. This demonstrates how, as the number of particles is increased, vertical layers are added when possible for the dome-first, while the layer grows horizontally with layer-first. The dome-first geometry is depicted upside-down in the side on view relative to part (**a**).

A demonstration of the charge measurement process shown in Fig. 2a,b. The system is set to rotate with particles in the charging capsule. The particles fall into the Faraday cup which measures their charge. They gain more charge during contact while falling into the Faraday cup, so that when they fall back out of the Faraday cup, the charge reading sees more charge leaving the cup than had initially entered it. This charge difference is the charge gained on contact with the Faraday cup. The particles will continue contact charging against the grounded container and the Faraday cup under continuous rotation until equilibrium charge is reached. Simultaneously, the charge on the particle is measured at each turn as it either falls into or out of the Faraday cup. A square wave signal in the electrometer charge reading describes the charge build-up, as demonstrated by the solid line in Fig. 2b. The charge per particle is extracted by dividing the difference in charge measurement before and after the particles fall into/out of the Faraday cup by the total number of particles.

### Electrical breakdown model

Electrical breakdown of air the process whereby electrons generated by natural ionisation in air are accelerated enough to cause an avalanche of ionisations through impact with air molecules. This requires an electric field of ~ 3 MV/m at atmospheric pressure^50^. This required field strength reduces at first as the pressure reduces, due to the increase in the mean free path of an electron in low pressure air. The Paschen curve shows that there is a critical pressure at which the electric field strength required to ionise air is at a minimum^51^. The dielectric strength of air increases again below this pressure as a vacuum is approached, due to the low number density of molecules in the air. The mean free path of an electron will get shorter above this critical pressure, requiring a stronger electric field for ionisation on impact with an air molecule.

Full spark discharge only occurs in a uniform electric field between conducting electrodes. The presence of static charge on insulators, and curvature on charged surfaces, lead to non-uniform electric fields which cause localised ionisation in smaller regions close to the charged surface. These non-uniform fields cause corona or brush discharge.

We consider the electric field at the surface of a spherical insulating particle next to a grounded flat conductor. The electric field is strongest near the curved surface of the particle, which is where partial discharge will occur if the charge is high enough. Peek’s law^52^ has been used to describe corona breakdown around high voltage wires^53^. The law is an empirical relationship relating the wire radius $r$ to corona onset electrical field strength ${E}_{\text{S}}$:

1 $${{E}}_{\text{S}}={{E}}_{0}\updelta \left(1+\frac{{c}}{\sqrt{{\delta r}}}\right).$$

${E}_{0}$ and $c$ are empirical constants. $\delta$ is the air density factor which is the ratio $P/{P}_{\text{atm}}$ of air pressure $P$ to atmospheric pressure ${P}_{\text{atm}}$. Peek’s law shows that the breakdown electric field strength at the surface of a conducting wire is inversely proportional to the square root of its radius, and that it approaches a constant ${E}_{0}$ the surface of the conductor becomes flat. The air density factor accounts for the fact that the dielectric strength of air decreases as pressure decreases. It does not account for the increase in dielectric strength below the critical pressure.

Peek’s law can be used for the geometry of conducting spheres next to a grounded flat conductor^53–55^. Here we adapt it to predict the saturation surface charge density ${\sigma }_{\text{sat}}$ on a spherical particle due to electrical breakdown. The maximum electric field strength between a spherical particle and a flat conducting surface will be experienced just as the surfaces separate. The electric field strength ${E}_{\text{S}}$ at the point of the surface closest to the conducting surface can be calculated using the method of images and is given by ${E}_{\text{S}}=2\sigma /{\varepsilon }_{0}$, where $\sigma$ is the surface charge density of the particle. Equating this expression with Eq. (1) yields the following expression for the maximum possible surface charge density on a spherical particle triboelectrically charging against a flat grounded conducting surface before electrical breakdown occurs:

2 $${\upsigma }_{\text{sat}}\left({r}\right)=\frac{{\upvarepsilon }_{0}{{E}}_{0}\updelta }{2}\left(1+\frac{{c}}{\sqrt{{\delta r}}}\right).$$

The non-uniform electric field at charged curved surfaces can explain the inverse radius dependence of this equation^50^. The electric field generated at a curved surface will decrease quickly as the distance above the surface increases, and the rate of decrease is inversely related to the radius of curvature. This inverse dependence between radius of curvature and electric field gradient holds true also for insulating particles, meaning that Peek’s law can also be used to approximate the surface charge density required on insulating particles of varying size to cause electrical breakdown. The distance over which the electric field is strong enough to cause ionisation will not be large enough if the electric field at the surface just exceeds the electrical breakdown limit. Any electrons arising from natural ionisation in this region do not gain enough energy to cause further ionisation of air. A stronger electric field is necessary at a curved surface in order to ensure that the region in which the electrical breakdown limit is exceeded is large enough for electrons to gain enough energy to cause ionisation.

The surface charge density of an insulating particle will be non-uniform as charge builds up. If local hotspots of charge can cause corona discharge, this can lead to a mosaic of positive and negative surface charge patches, where the combined electric field generated is just below ${E}_{\text{BD}}$^48^. Gauss’ law cannot be used to calculate the electric field generated by an insulating particle if this is the case, as it relies on the surface charge density being uniform. Non-uniform charge density gives rise to tangential electric field components that do not cancel out at the surface of the particle. Local electric fields can thus be stronger, exceeding the value that would be calculated using Gauss’ law and the total charge on the particle. This deviation from Gauss’ law has implications for experimental measurements, where the electric field cannot be calculated accurately based on the measured total charge on an insulating particle. The true electric field at the surface will be higher than that calculated using Gauss’ law, and the difference will be relative to the non-uniformity of the surface charge density. As a result, it is expected that the electric field calculated based on experimental charge measurements will follow a trend similar to Eq. (2) but will be shifted down to a lower magnitude.

### Triboelectric charge saturation on single particles in air and vacuum

**Figure 3:**
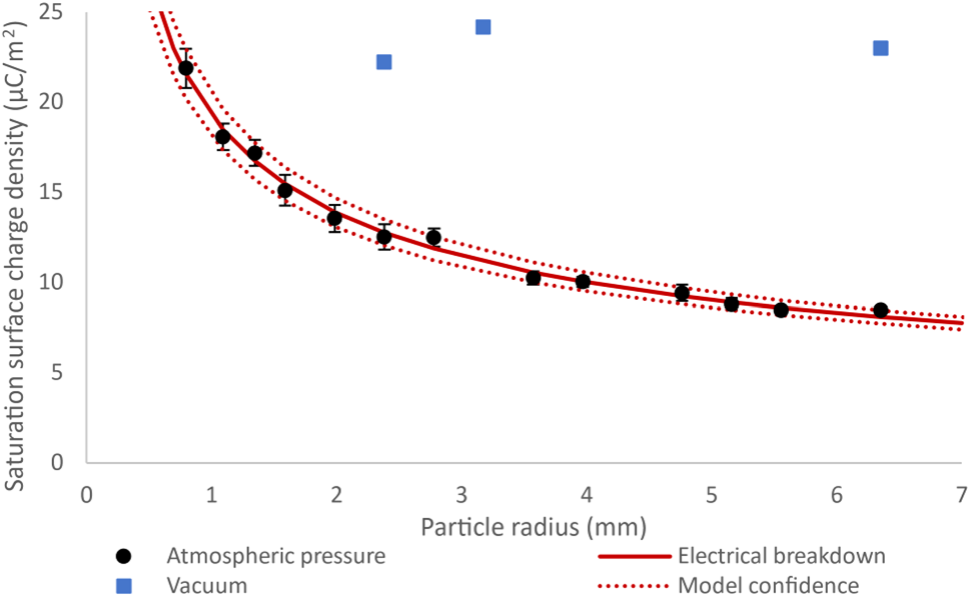
Absolute value of saturation surface charge density of PTFE spheres as a function of particle radius when triboelectrically charged against stainless steel. The circular datapoints are experimental measurement taken at atmospheric pressure, and the square datapoints at 4 × 10^–4^ mbar. The solid line is the best fit of the electrical breakdown model from Eq. (2), whose 95% confidence-band is enclosed between the dotted lines. All confidence intervals were calculated using a 95% t-test. The R^2^ value for the model is 0.993.

The effect of particle size on saturation charge for single particles was investigated primarily using PTFE spheres of diameter ranging from 1.59 to 12.70 mm. All particles were charged to saturation against grounded stainless steel at atmospheric pressure. The environment was kept constant at 30% relative humidity (RH) and 30 °C. The experiments were repeated in a vacuum at 4 × 10^–4^ mbar using the 4.76, 6.35, and 12.7 mm particles. The charge measured under vacuum was observed to increase steadily before suddenly ceasing to be registered by the electrometer at a cut-off point. This cut-off was suspected to be due to electrostatic adhesion and is investigated in more detail later. The cut-off charge of the particles was measured in order to compare the maximum observed charge under vacuum with the saturation charge measured in air. The saturation surface charge density from the experimental measurements is shown in Fig. 3. The electrical breakdown model from Eq. (2) which is based on a modified Peeks law is fit to the experimental data and shown with the solid line in order to verify that the saturation values follow the expected trend. The parameters ${E}_{0}$ and $c$ are fit to 0.168 and 0.788 respectively, and the R^2^ value is 0.993. The dotted lines enclose the model’s 95% confidence interval.

There is excellent agreement between the electrical breakdown model and the measured saturation surface charge density of the PTFE particles at atmospheric pressure. The charge of the particles in a vacuum is much higher than at atmospheric pressure. The expected trend is seen at atmospheric pressure, and repeating the same experiment in a vacuum where the dielectric constant of the surrounding medium is higher leads to a much higher observed charge. This suggests that electrical breakdown is most likely the charge saturation mechanism.

**Figure 4:**
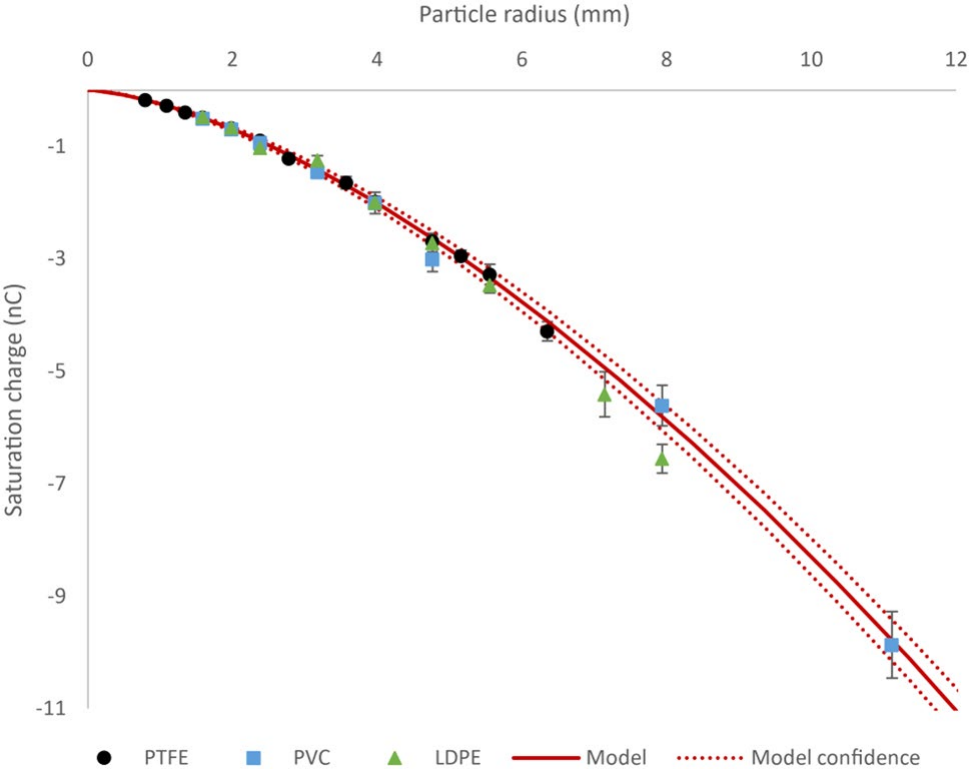
The saturation charge on PTFE (circles), PVC (squares), and LDPE (triangles) particles as a function of particle radius when triboelectrically charged against grounded stainless steel. The solid line is the saturation charge at which electrical breakdown occurs calculated based on the model from Eq. (2), which was fit to the PTFE saturation charge data in Fig. 3. The dotted lines enclose the model’s 95% confidence band.

The experiments were repeated in air using different polymer materials to provide further evidence that electrical breakdown is the mechanism responsible for charge saturation. Particles of different material are expected to saturate as predicted by Eq. (2) if electrical breakdown is the saturation mechanism, despite there being a different effective work function difference between the materials triboelectrically charging. LDPE and PVC particles of 3.18–15.88 mm and 3.18–22.23 mm diameter respectively were used. Figure 4 shows the charge measured on the particles as a function of radius. All confidence intervals were calculated using a 95% confidence t-test. The solid line is the electrical breakdown model from Eq. (2) which was fit to just the PTFE data as shown in Fig. 3. The dotted lines enclose the model’s 95% confidence band.

The experimentally measured saturation charges on the PVC and LDPE particles align closely with the electrical breakdown model which was fit to the PTFE data. In addition to the increased charge under vacuum observed in the previous section, this agreement between the model and multiple materials further confirms that electrical breakdown is the saturation mechanism for single polymeric insulating particles in air. The model also yields accurate predictions for saturation charge for sizes that the model was not fitted to. This is evident from the accurate prediction of the saturation charge on the 22.23 mm diameter PVC particle for example, which is almost twice as large as the largest of the PTFE particles.

### Multiple particle electric field simulations

A simulation of the charging process was developed to determine whether electric field build-up is the saturation mechanism for charge in multiple-particle systems. Figure 2a shows a schematic of the automated experimental charging process used. A group of particles are placed in a grounded conducting charging capsule which is rotated clockwise. The particles roll down the side of the capsule, charging triboelectrically. As the particles reach the end of the slope they agglomerate in the corner, before spreading out along the end of the capsule as an inversion is completed.

Here we consider two primary geometries for simulation as shown in Fig. 2a,c: dome-first and layer-first. Dome-first geometry simulates the agglomeration or dome of particles in the corner of the charging capsule during rotation. At this point in the rotation the particles are tightly packed and the total electric field at the centre particle is greatest. This is the point at which further charge build-up would be prevented due to electrostatic discharge from dielectric breakdown of air. Next, layer-first geometry simulates the adhesion of particles to the grounded charging capsule due to the Coulomb force between particles and image charges. A strong enough Coulomb force prevents further triboelectric charging. This would manifest as a flat layer of particles on the bottom surface of the capsule, as any particles in a potential second layer would be repelled by its neighbours that are adhering to the capsule wall.

The maximum electric field for both geometries is calculated by considering the closest packing possible using a hexagonal lattice. Dome-first geometry is simulated by adding one particle at a time until a complete hexagonal layer is formed, then creating new layers of particles on top until a complete dome is made using hexagonal close packing. With layer-first geometry, a single hexagonal packed layer of particles is built-out until a maximum diameter is reached instead of creating new layers of particles vertically. Both the dome-first and layer-first geometries are shown in Fig. 2c. The maximum layer diameter was set to 60 mm, the diameter of the charging capsule used in the experimental tests.

**Figure 5:**
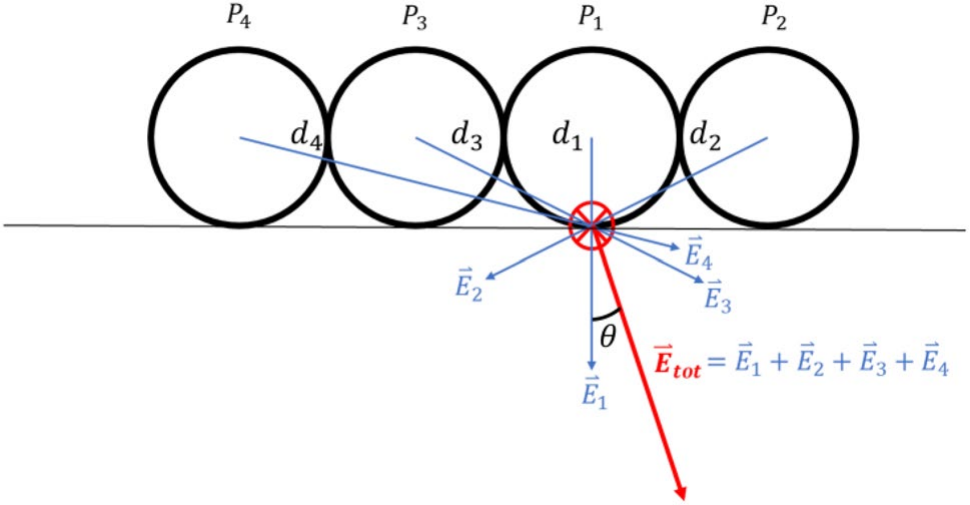
Schematic of the electric field calculation for a collection of particles. Four particles $${P}_{i}$$ are displayed in a row. The electric field is calculated at the contact point between the centre-most particle and the charging capsule wall. The particles are assumed to be point charges at the centre of each particle, a distance $${d}_{i}$$ from the contact point where the electric field is calculated. The total electric field $$ {\overset{\lower0.5em\hbox{$\smash{\scriptscriptstyle\rightharpoonup}$}}{E} _{{{{tot}}}} } $$ is the vector sum of the electric field $$ {\overset{\lower0.5em\hbox{$\smash{\scriptscriptstyle\rightharpoonup}$}}{E} _{{{{i}}}} } $$ created by each particle $$i$$ at the contact point. The direction of the total electric field makes an angle $$\theta $$ to the normal vector pointing into the surface of the charging capsule.

The magnitude and distribution of the electric field created by a group of particles determines whether dielectric breakdown of air occurs, or if the Coulomb force is strong enough for particles to adhere to the grounded wall. In these simulations the electric field was calculated at the contact point between the centre particle in the lattice and the charging capsule, assuming the surface charge density of the particles is uniform. The total electric field ${E}_{\text{tot}}$ at the point of contact is the sum of the electric field created by each particle and its associated mirror image on the wall of the grounded capsule:

3 $$ E_{{{\text{tot}}}}  = \left| {\overset{\lower0.5em\hbox{$\smash{\scriptscriptstyle\rightharpoonup}$}}{E} _{{{\text{tot}}}} } \right| = 2\mathop \sum \limits_{{i = 1}}^{N} \frac{{k_{C} q_{i} }}{{d_{i}^{2} }}, $$

where $N$ is the total number of particles, ${k}_{C}$ is the Coulomb constant $1/4\pi {\varepsilon }_{0}$, ${q}_{i}$ is the charge on particle $i$, and ${d}_{i}$ is the distance from the centre of particle $i$ to the contact point between the centre particle in the geometry and the charging capsule as depicted in Fig. 5. In reality the surface charge density on the surface of insulating particles is not constant, but stochastic in nature, and thus Eq. (3) is not a perfect representation of the electric field generated by a group of insulating particles. We decide to use it here however, as we are considering the saturation of charge on the surface of the particles as it approaches an equilibrium point, and wish to predict the trend of particle number dependence and the charge required to cause electrical breakdown.

Equation (3) represents the magnitude of the total electric field at the point of contact between the centre particle and the capsule wall. This value for the electric field is used to determine whether the dielectric strength of air (~ 3 MV/m^56^) is being exceeded, leading to a cessation of further charge build-up. Only the normal component ${E}_{\text{N}}$ of the electric field is considered when checking whether a particle adheres to the capsule wall:

4 $$ E_{N} = \left| {\overset{\lower0.5em\hbox{$\smash{\scriptscriptstyle\rightharpoonup}$}}{E} _{{{{N}}}} } \right| = E_{{{\text{tot}}}} \cos \theta , $$

where $$\theta $$ is the angle between the total electric field vector $${\overset{\lower0.5em\hbox{$\smash{\scriptscriptstyle\rightharpoonup}$}}{E} _{{{\text{tot}}}} }$$ and the normal vector pointing into the surface of the charging capsule. Both Eqs. (3) and (4) are applied to the 3D dome-first and layer-first geometries such as those in Fig. 2c to calculate the total and normal electric field strengths at the centre of groups of particles laid flat on a surface or congregated in a corner.

### Triboelectric charge saturation on multiple particles in air

PTFE spheres of 6.35 and 12.7 mm diameter were tribocharged in batches of 1 to 64 and 1 to 32 particles. A batch refers to the number of particles charged together at the same time. Each batch size was charged for 300 rotations of the charging capsule, and this was repeated 11 times, yielding over 1600 charge measurements per batch size. More information about the charge measurement is given in Note 1 of the Supplementary Information.

The maximum batch sizes of 64 and 32 for 6.35 and 12.7 mm particles is create 1–1.5 layers of particles at the base of the charging capsule. The small number of layers, combined with the 300 experimental rotations, suggest that most, if not all, particles will be in contact with the long side of the charging capsule as it rotates as shown in the schematic in Fig. 2a. The batches will reach maximum heights of 32.3 and 48.7 mm respectively in the dome-first geometry, remaining contained in one half of the charging capsule when congregating in a corner on rotation.

**Figure 6:**
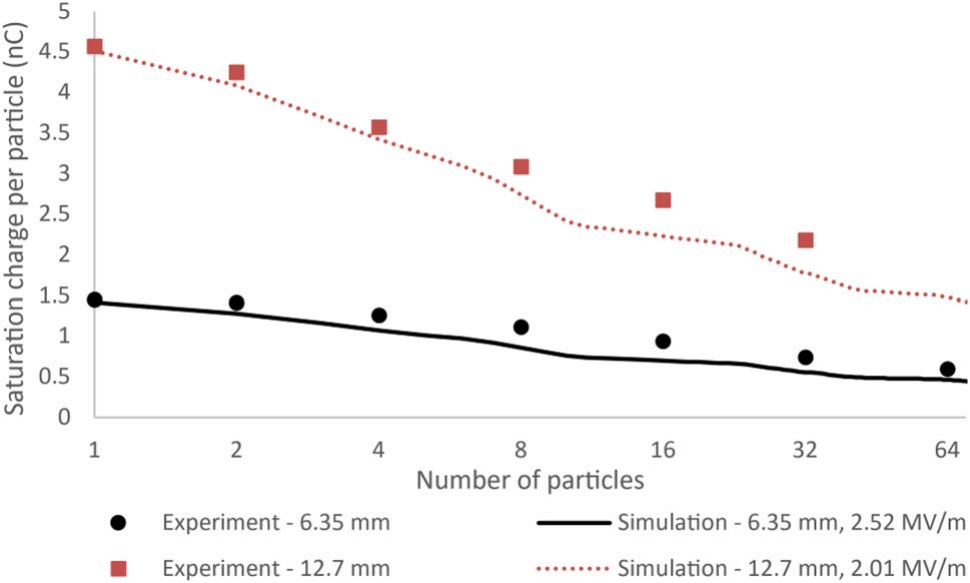
Absolute value of experimental and simulated triboelectric charge saturation as a function of number of particles. The figure shows batches of 6.35 and 12.7 mm diameter PTFE particles in batches of 1–64 particles. The circular and square data points represent the experimental results for the 6.35 and 12.7 mm diameter particles respectively. The solid and dotted lines are simulations made using the dome-first model with particle sizes of 6.35 and 12.7 mm, and breakdown electric field strengths of 2.52 and 2.01 MV/m respectively. The x-axis uses a base 2 logarithmic scale. Experimental datapoints are the average of over 1600 individual charge measurements taken over 12 charging runs, which is described in more detail in the Supplementary Information Note 1.

Figure 6 shows the absolute value of the saturation charge on the particles as a function of batch size, along with dome-first model simulations which predict the saturation charge due to electrical breakdown. Equation (3) was applied to particles in the dome-first geometry to predict the minimum charge per particle at which electrical breakdown can occur. The circular and square datapoints show the experimental data for 6.35 and 12.7 mm diameter PTFE particles respectively, while the solid and dotted lines show the electrical breakdown simulations. The x-axis is the number of particles in a batch and has a base 2 logarithmic scale.

The data in Fig. 6 suggests a logarithmic relationship between saturation charge per particle and the number of particles charged in a batch. Furthermore, the dome-first simulation is well aligned with the experimental data. The difference in breakdown electric field strength between the two particle sizes is due to the effect of particle size on the local electric field gradient as was seen for single particle charging.

The simulations match the observed logarithmic drop-off in charge per particle as the number of particles increases. The simulations match the single-particle experimental charge, while predicting a slightly smaller charge per particle for multiple particles. This is due to the simulations considering the closest packing possible, yielding a lower limit for saturation charge. Any variations from the simulation in particle dynamics and packing geometry, which are expected in the experiments, would require a larger electrical breakdown-inducing charge than the simulations predict.

**Figure 7:**
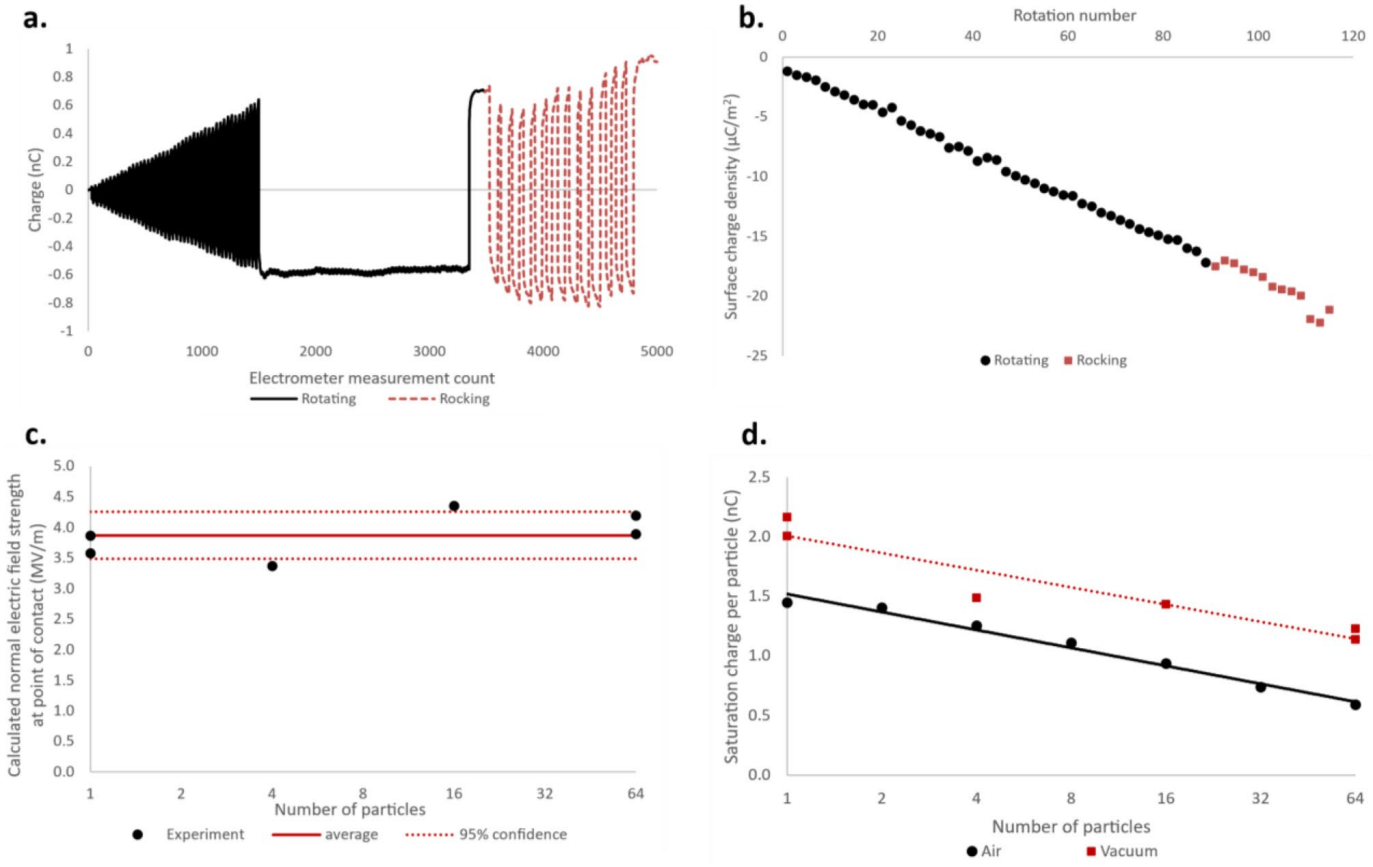
The electrostatic adhesion of charged particles in vacuum. (**a**) Electrometer charge reading for a 4.76 mm diameter PTFE particle as the charging capsule rotates back and forth by 180°, building up triboelectric charge on the particle until it adheres to the Faraday cup wall due to the Coulomb force. This rotating motion is represented by the solid line. The dashed line shows the charge reading after the capsule begins rocking side to side after each 180° rotation. Here the particle is dislodged, gaining more charge until the Coulomb force becomes strong enough to overcome the rocking motion. (**b**) The surface charge density on the 4.76 mm particle as a function of capsule rotations. The circular and square points are calculated from the rotating and rocking regimes respectively in part (**a**). The particle stuck to the Faraday cup surface at the final circular datapoint and became unstuck and resumed charging from the first square datapoint. (**c**) The calculated normal component of the electric field at the centre of groups of particles, using the layer-first model. The datapoints were calculated using experimental values for batches of 1, 4, 16, and 64 6.35 mm diameter particles. The average electric field is shown as a solid line, and the 95% confidence range calculated using t-test is shown as dotted lines. (**d**) The saturation charge on batches of 6.35 mm PTFE particles in vacuum and air with the logarithmic lines of best fit. The charge is considerably higher in vacuum, leading to electrostatic adhesion. The x-axes in (**c,d**) are base 2 logarithmic scale.

These results provide evidence that electrical breakdown of air is the mechanism causing charge to saturate at lower values as the number of particles increases. The experiments were repeated under vacuum in order to further test this hypothesis. It was found that particles gain enough charge to adhere to the side of the charging capsule under vacuum, preventing further charge build-up. Figure 7a shows the electrometer charge reading while a 4.76 mm PTFE particle is being charged at a pressure of 4 × 10^–4^ mbar.

The charging capsule is initially set to rotate back and forth by 180°, represented by the solid line in Fig. 7a. The charge reading stops at about 1500 counts, with the particle seeming to remain lodged in the Faraday cup. The system is set to rock back and forth quickly in between 180° rotations from about 3500 counts, this is shown by the dashed line in the figure. Charge measurement restarts almost instantly after the system starts rocking. At about 4800 counts the particle becomes stuck, having gained enough charge for the coulomb force to overcome the rocking motion.

Figure 7b shows the surface charge density of the particle from Fig. 7a as a function of number of capsule rotations. The charge increases steadily in the rotating phase (circular datapoints) until electrostatic adhesion causes it to stop. The square datapoints show the charge measured in the rocking phase, which picks up right after the rotating phase, showing the charge measured increasing at the same rate.

The charge at which particles adhered to the charging capsule was measured experimentally for batches of 1, 4, 16 and 64 particles of 6.35 mm diameter. This experimental charge was then used to calculate the normal electric field strength at the contact point with the capsule wall using the layer-first model and Eq. (4). This calculated cut-off electric field strength is shown in Fig. 7c as a function of the number of particles being charged in a batch. The average field strength is shown as a line, along with its 95% confidence interval calculated using a t-test.

The data in Fig. 7c shows that the cut-off normal electric field strength at which particle charge stops being measured is constant at about 3.87 ± 0.39 MV/m. This constant cut-off normal electric field strength confirms that particle adhesion to the charging capsule is taking place, corresponding to the electric field needed to create a strong enough Coulomb force under these particular experimental conditions. It was found that adhesion occurs when the Coulomb force attracting the particles to the capsule wall was between 1.5 and 2.9 times the gravitational force acting on the particles.

Figure 7d shows the adhesion charge in vacuum as a function of number of particles, along with the saturation charge of the same particles in air. The data clearly shows that larger charge can be reached in a vacuum than in air. The charge reached under vacuum before electrostatic adhesion to the charging container was ~ 30% greater than the saturation charge reached in air for all batch sizes. This supports the conclusion that electrical breakdown of air is the saturation mechanism for batches of multiple insulating particles being triboelectrically charged in air.

The data in Fig. 7 shows that electrostatic adhesion is causing charge to stop accumulating in a vacuum. This is dependent on the cumulative electric field of groups of particles, similarly to electrical breakdown for particles charging in air. The dependence of both of these mechanisms on electric field strength would explain why the saturation charge data for air and vacuum in Fig. 7d both have the same trend, despite being determined by different saturation mechanisms.

### Electrostatic charge mitigation at low pressure

The dielectric strength of a gas will decrease as its pressure decreases from atmospheric pressure towards a vacuum. The dielectric strength will then asymptotically approach infinity beyond a critical pressure as vacuum is approached. If, as the results in earlier sections of this paper suggest, electrical breakdown of air is the saturation mechanism for charge on polymer particles, it should therefore be possible to reduce the charge held by materials by maintaining them under low pressure. This ability to remove the charge from materials, even while being rubbed together, would have numerous applications in industrial processes, from powder transport to dust mitigation from surfaces. Observing this behaviour, in line with the prediction based on the model in Eq. (2), would provide further evidence that electrical breakdown is the charge saturation mechanism for insulating polymeric particles.

**Figure 8:**
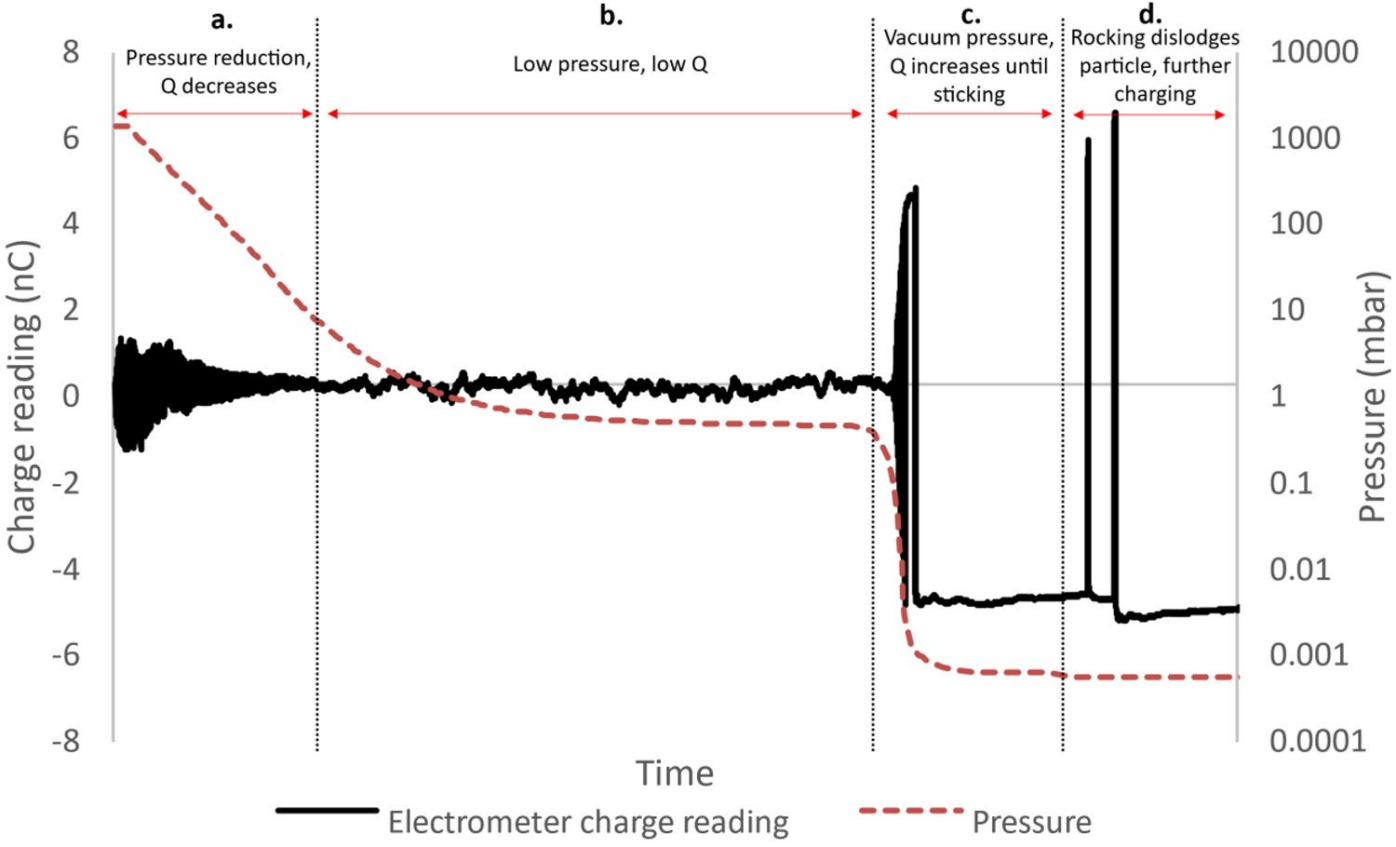
The electrometer charge reading (solid line) and pressure (dashed line) as a function of time as a 12.7 mm diameter PTFE particle is charged in the automated charging system. The particle was constantly charged as the automated system rotated back and forth by 180°. The pressure in the chamber was reduced from atmospheric pressure to a vacuum of ~ 4 × 10^–4^ mbar. The figure is split into 4 sections describing the effect of pressure on the particle’s charge: (**a**) The charge on the particle reduces as the chamber pressure reduces. (**b**) The charge on the particle is approximately zero in the low pressure region from ~ 0.1 to 10 mbar. (**c**) The turbo pump was turned on after the roughing pumps reached steady state pressure. The charge on the particle quickly increases below 0.1 mbar pressure. The charge reading stops when the particle adheres to the Faraday cup due to the Coulomb force. (**d**) The charging system begins rocking back and forth between each 180° rotation, dislodging the adhered particle, and leading to further charging and charge measurement. The increased charge is enough to overcome the dislodgment due to rocking.

An experiment was performed on a single 12.7 mm diameter PTFE particle, where air pressure was reduced from atmospheric pressure to 4 × 10^−4^ mbar while the particle was continuously triboelectrically charged. The electrometer charge reading for this experiment is shown along with the air pressure in Fig. 8. The solid line represents the electrometer charge reading in nanocoulombs which given on the primary lefthand y-axis. The pressure reading is represented by the dashed line and is given on the secondary righthand y-axis. The x-axis represents time, and both charge and pressure readings have been aligned based on their timestamps. The entire experiment spanned 100 min.

The data in Fig. 8 has been split into 4 sections, outlining the effect of pressure on charge saturation. Figure 8a shows the initial reduction in pressure from atmospheric pressure down to ~ 10 mbar. The charge on the particle is given by the amplitude of the solid line, in effect its vertical thickness. The charge on the particle can be seen to gradually reduce along with pressure towards a small, near negligible level. This reduction in saturation charge with pressure is in line with the electrical breakdown model from Eq. (2). The mean free path of an electron in air increases with reducing pressure, meaning that a weaker electric field can impart enough energy on an electron to cause ionisation of air molecules on contact.

Figure 8b shows the low pressure, low charge region between 0.1 and 10 mbar. The charge on the particle remains constant at near-zero in this region, as the dielectric strength of the low pressure air is very low.

Figure 8c shows the reduction of pressure from ~ 0.1 to ~ 0.0004 mbar. Here the charge on the particle increases quickly to a much higher level than at atmospheric pressure, before adhering to the inside of the Faraday cup due to the Coulomb force.

Figure 8d shows the introduction of rocking back and forth between each 180° rotation of the charging capsule. This rocking back and forth dislodged the particle 4 times, leading to two further charge measurements, where the charge is increased relative to the sticking charge during simple rotation as seen in Fig. 8c.

**Figure 9:**
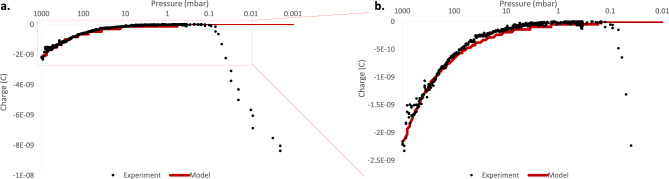
The triboelectric charge on a 12.7 mm diameter PTFE particle in air as a function of pressure. (**a**) Shows the pressure range from 0.001 to 1000 mbar. The circular datapoints represent the experimental datapoints calculated by taking the amplitude of the square wave from Fig. 8. The solid line is the electrical breakdown model as a function of pressure from Eq. (2). (**b**) A magnification of (**a**) focusing on the pressure range from 0.01 to 1000 mbar. The reduced scale allows for better comparison of the model to the experimental data in the low pressure range.

The data in Fig. 8 clearly shows the charge on the particle to reduce with reducing pressure before suddenly increasing below a certain level. This is consistent with the electrical breakdown saturation mechanism. The charge values at each turn were extracted and plotted against pressure in Fig. 9. The electrical breakdown model from Eq. (2) was overlaid on the data as a solid line. Figure 9a shows the entire set of datapoints from 1 × 10^–3^ mbar to atmospheric pressure, while Fig. 9b shows a magnified view of the data from 0.01 mbar to atmospheric pressure.

Figure 9a clearly shows the charge on the particle reducing towards zero with reducing pressure before increasing to a much higher level as a vacuum is approached. The magnification in Fig. 9b more clearly shows the comparison of the experimental results with the electrical breakdown model from Eq. (2). There is very good alignment between the model and the experiments as the pressure reduces from atmospheric pressure to ~ 0.1 mbar. This alignment between model and experiment further consolidates the conclusion that electrical breakdown is causing charge saturation on the polymer particles.

The model breaks down in the vacuum pressure region, not predicting or accounting for the sudden increase in charge below a certain pressure. An update to the model predicting this transition point is a promising area of future work. The behaviour is very similar to the Paschen curve, however Paschen considers complete electrical breakdown between flat conducting plates, while the geometry of the electric field between an insulating sphere and a conducting surface is more complex.

The results in Fig. 9 show that it is possible to almost discharge particles completely by maintaining them in a low pressure environment, as long as the pressure is not low enough that electrical breakdown can no longer occur. The processes of brushing dust off a surface or transporting powder in industrial processes involve mechanical agitation of particles, causing many contacts and subsequent charge transfer, worsening electrostatic adhesion of the particles to surfaces, or interparticle cohesion. As such, this ability to control the charge on particles, and to keep it close to zero despite repeated contact, holds great potential for dust mitigation and material transport applications.

## Methods

### Materials

PTFE polymer spheres were obtained from the Plastic Ball Company (UK). In these experiments, particles of 6.35 and 12.70 mm diameter were used in batches ranging from 1 to 64 particles.

### Custom Faraday cups

It was necessary to build a custom Faraday cup with the correct geometry and cup material while designing an automatic charging and measurement system. Cups of any geometry or conducting material can be used for construction, as Faraday cups works by charge induction.

For these experiments, dimensions of 85 mm diameter × 85 mm height were used for the outer cup, and 60 mm diameter × 60 mm height for the inner cup. All cups used were made of 304 stainless steel, polished to a 0.4 μm finish (Pharma Hygiene Products COC4085P UK).

To construct the Faraday cup a 1 cm hole was drilled in the side of the outer cup. A 75 Ω panel mount BNC connector was mounted on the drilled hole (RS pro 546-4904 UK). A 1 cm thick sheet of PTFE was cut out and placed at the bottom of the outer cup, on which the inner cup was mounted. The tip of the BNC connector was soldered to the surface of the inner cup using lead free silver solder (RS pro 756-8884 UK) and S26 fluid flux (Solders & Fluxes UK). The space between the cups was then filled with an electrically insulating polyurethane potting compound (RS pro 199-1395 UK). Immediately after potting, the cups were placed in a vacuum for 30 min to off-gas any bubbles in the compound. This is done to improve the resistive properties of the potting compound, and to ensure trapped bubbles do not expand and compromise the structure when the cup is used in a vacuum chamber.

### Environmental control

Experiments done in air were done under controlled humidity and temperature using a sealed glovebox. To control humidity, the glovebox was connected to two desiccant capsules via an air tube and a pump. This pump pulled air from the chamber, passing it through the desiccant capsules to dry it, before being pumped back into the main chamber. In addition, the chamber was connected to a feed of dry nitrogen gas which could be used to purge humid air. There were a number of valves attached to the chamber so that air would be forced out of the chamber with the introduction of nitrogen, maintaining ambient air pressure inside. Using nitrogen purging and desiccant, the humidity in the glovebox could be controlled in the range of 5–95%. For temperature control, a PID controlled heater was used in conjunction with a chiller. This allowed for temperature control from 12 to 60 °C.

### Vacuum control

A vacuum chamber connected to three roughing pumps and two turbo pumps was used to perform charging experiments under low pressure and at vacuum. A high vacuum of 3 × 10^–4^ mbar was achievable using this system. The automated charging system was placed in the vacuum chamber, and the BNC cable connecting the electrometer to the Faraday cup was fed in using a vacuum rated feedthrough (LewVac FH-BNC-D-40CF UK). The Arduino board was kept in the vacuum chamber, however the USB cable connecting to the computer to power and control the board was also separated and fed through using a custom-made electrical feedthrough. The vacuum chamber was fit with a built-in pressure transducer which was accurate at in the high vacuum–low pressure range (< 25 mbar). An additional pressure transducer (Omega PXM409-001BVUSBH UK) was mounted on the side of the vacuum chamber which was accurate in the low pressure–atmospheric pressure range. Combined, the two calibrated transducers allowed for a continuous logged pressure reading as air was pumped out of and reintroduced into the chamber.

### Supplementary Information

Supplementary Information.

## Data Availability

Experimental data and simulation results are available via the Harvard Dataverse repository at 10.7910/DVN/INOCFX. All custom computer codes that support the findings of this study are available from the corresponding author upon reasonable request.
