# Supplementary material for: Triboelectric charge saturation on single and multiple insulating particles in air and vacuum
Source: Sci Rep. 2023 Sep 13;13:15178. doi: 10.1038/s41598-023-42265-0 (PMC10499910; doi:10.1038/s41598-023-42265-0)
Supplement: Supplementary file 1 — Supplementary Information. [file 41598_2023_42265_MOESM1_ESM.pdf]

Supporting note 1 – Measuring charge saturation for multiple particles

PTFE spheres of 6.35 and 12.7 mm diameter were tribocharged in batches of 1 to 64 particles using the automatic charging system described in the main manuscript. Supplementary figure S1 shows the charge per particle measurements for the entire charging runs for all batches and both particle sizes. Each charging run was repeated 12 times, where the datapoints are the average of the 12 measurements for that batch at a certain rotation number of the automated charging system. The confidence intervals were calculated using a 95% t-test.

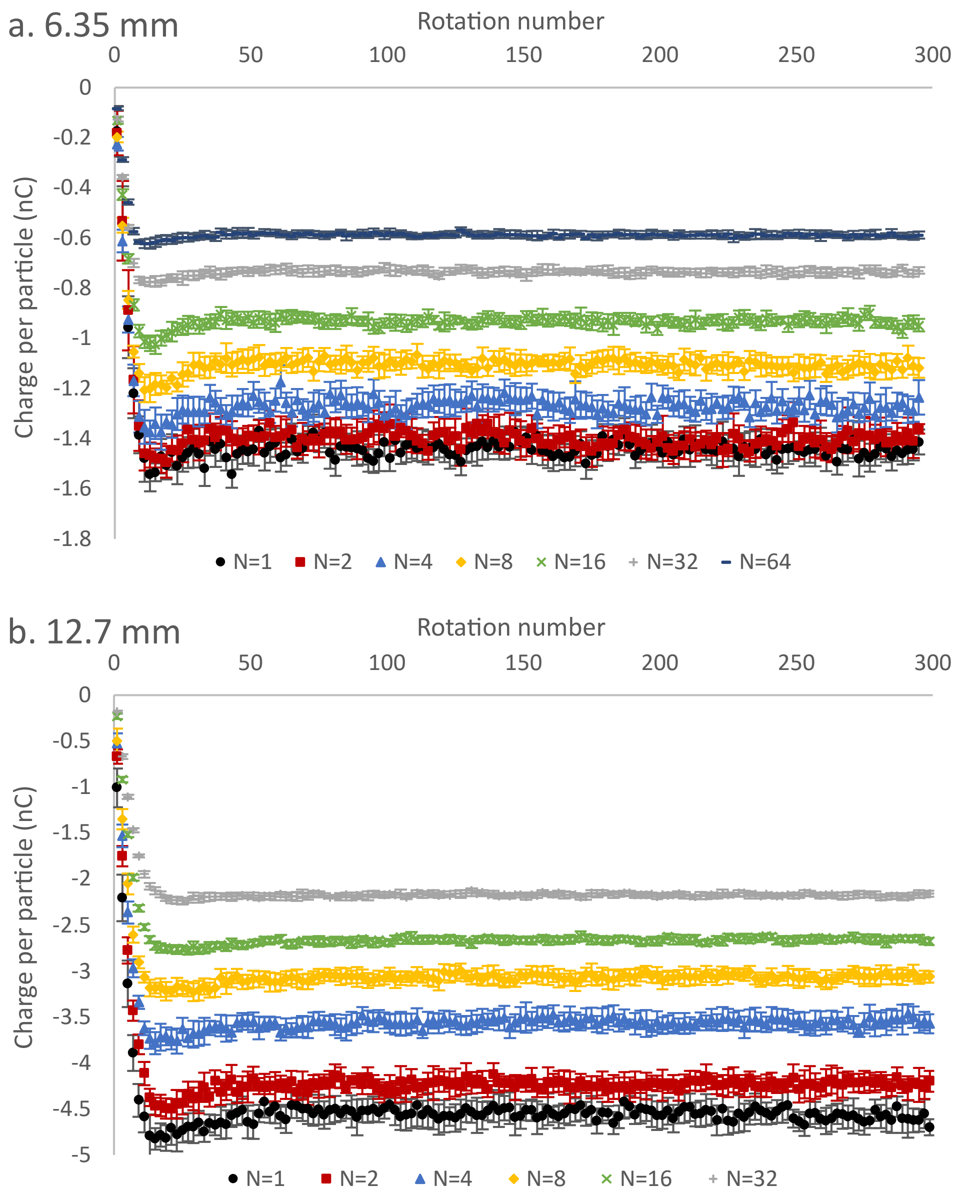

Supplementary figure S1: Charge per particle on batches of (a) 6.35 and (b) 12.7 mm diameter PTFE particles as a function of rotation number of the automated charging system. Each charging run represents a batch of a certain number of particles and was repeated experimentally 12 times. The datapoints are the average of the 12 repeats, and the confidence intervals were calculated using a 95% t-test based on the 12 measurements for a certain rotation number.

Supplementary figure S1 clearly shows that the saturation charge per particle decreases as the number of particles in a batch increases. The charge measured on the particles can be seen to initially increase from a low initial value and level off at a saturation point. The saturation charge was calculated by taking the average of all datapoints for a certain batch after the curve levels off.
